# Whole‐body glucose tolerance is impaired in male and female congenic mice lacking nicotinamide nucleotide transhydrogenase (NNT)

**DOI:** 10.14814/phy2.71167

**Published:** 2026-11-03

**Authors:** Jinmeng Yang, Jingyi Song, Jo‐lene de Deugd, Marcel Jaklofsky, Georgios Nikolopoulos, Melissa Bekkenkamp‐Grovenstein, Claudia Carmone, Jaap Keijer, Heather L. Petrick, Sander Grefte

**Affiliations:** ^1^ Human and Animal Physiology Wageningen University Wageningen Gelderland the Netherlands

**Keywords:** adipose tissue, aging, glucose homeostasis, insulin resistance, skeletal muscle

## Abstract

Nicotinamide nucleotide transhydrogenase (NNT) is a mitochondrial redox protein. Previous reports of NNT deficiency impairing glucose tolerance in young male mice were potentially confounded by background strain differences. This study investigated whether an NNT deletion specifically in a congenic mouse model induces glucose intolerance across sexes and ages. Indirect calorimetry, oral glucose tolerance tests, and tissue analyses were performed in adult (6‐month‐old) and old (24‐month‐old) male and female congenic mice with (*Nnt* ^*WT*^) and without (*Nnt* ^*Δ*^) NNT (*n* = 8–15). While older mice exhibited increased body weight and higher fasted insulin levels (*p* < 0.0001), NNT deficiency did not affect these parameters, energy expenditure, or substrate utilization in either sex. Following glucose ingestion, glucose tolerance worsened in *Nnt* ^*Δ*^ mice of both sexes. Glucose‐stimulated serum insulin was unaffected in adult females, but was lower in 24‐month *Nnt* ^*Δ*^ females (*p* = 0.009) and 6‐month *Nnt* ^*Δ*^ males (*p* = 0.0303). However, there were no effects of genotype on glucose‐stimulated signaling pathways (including PI3K, STAT3, phosphorylated‐AKT, sarcolemmal GLUT4 localization) in skeletal muscle (*p* > 0.295) collected 30 min following glucose administration. Similarly, glucose‐stimulated AKT phosphorylation ratio did not differ between genotypes within liver or mesenteric white adipose tissue (mWAT). Combined, congenic NNT deficiency induces whole‐body glucose intolerance in both male and female adult and old mice.

## 1 INTRODUCTION

Nicotinamide nucleotide transhydrogenase (NNT) is an enzyme located in the inner mitochondrial membrane and plays a key role in energy metabolism and antioxidant defense. Utilizing the proton motive force across the mitochondrial inner membrane, NNT generates NADPH which is essential for regenerating cellular antioxidant defense systems and maintaining redox balance (Francisco et al., 2022; Gan et al., 2024). As the presence of chronically elevated reactive oxygen species (ROS) is sufficient to impair whole‐body glucose handling (Houstis et al., 2006; Tangvarasittichai, 2015), there has long been interest in a potential role of NNT in regulating glucose tolerance.

The relationship between NNT and glucose metabolism was first discovered when the C57BL/6J sub‐strain, harboring an inactivating NNT mutation, was found to display impaired glucose tolerance compared to C57BL/6N lines retaining an intact NNT allele (Close et al., 2021; Kaku et al., 1988; Toye et al., 2005). This phenotype was primarily attributed to pancreatic β‐cell dysfunction and impaired insulin secretion (Close et al., 2021; Fergusson et al., 2014). Introducing functional NNT into the C57BL/6J strain effectively improved β‐cell dysfunction and glucose intolerance (Freeman et al., 2006). However, other research has indicated that the deficiency of NNT does not affect (Wong et al., 2010), or only moderately affects (Close et al., 2021) insulin secretion or glucose tolerance. As a result, the specific role of NNT in glucose homeostasis remains a controversial topic. A major problem underlying these diverse findings is the complex genetic background of C57BL/6J mice. Due to multiple other mutations in the genome, several other proteins besides NNT are not functional, which may influence glucose tolerance. Consequently, the limited use of congenic mouse models lacking solely NNT in most of the earlier literature makes these previous findings difficult to directly interpret (Ronchi et al., 2016), and it remains unknown whether NNT deficiency alone alters glucose tolerance and insulin resistance or secretion. In addition, existing literature on the role of NNT in glucose homeostasis has largely focused on males and early life stage in mice (Close et al., 2021; Toye et al., 2005). However, it is well established that female rodents are protected against glucose intolerance and insulin resistance (Pettersson et al., 2012), meaning the metabolic effects of NNT deficiency cannot be assumed to be identical in males and females. While it is well known that impaired glucose tolerance generally becomes more common with advancing age (Stout, 1994), it has also been reported that glucose tolerance does not deteriorate with age in C57BL/6J mice (Leiter et al., 1988), leaving the interaction between aging and NNT deficiency unclear. Combined, given the current contrasting findings, potential metabolic protection in females, and use of confounding non‐congenic mouse models, we aimed to investigate whether NNT deficiency differentially impacts glucose tolerance in both sexes at different ages.

To address this topic, we generated a congenic mouse model in our laboratory, harboring either an intact *Nnt* allele (B6JRcc(B6J)‐*Nnt* ^+^/Wuhap) or an *Nnt* deletion (BL6JRcc.BL6J‐*Nnt* ^*C57BL/6J*^/Wuhap). By utilizing this novel mouse model, we can attribute any observed differences between genotypes to NNT dysfunction rather than to the accumulated variants of a substrain, which addresses the principal interpretive limitation of the existing literature. The primary aim of this study was to determine if whole‐body deficiency of NNT impairs glucose tolerance in male and female mice, and if NNT deficiency compromises the typical age‐related metabolic stability. Assuming that the accumulated variants in previous models provided a compensatory effect, we hypothesized that mice lacking NNT would display impaired oral glucose tolerance, and this would be present in both sexes and be exacerbated in old age.

## 2 MATERIALS AND METHODS

### 2.1 Ethics statement and animals

All animal handling procedures were conducted in accordance with the EU Directive 2010/63/EU for animal experimentation and were approved by the Animal Welfare Committee of Wageningen University, Wageningen, The Netherlands (2020.W‐0019.004 and 2022‐W‐0033.004) and were performed in strict accordance with the ARRIVE guidelines. Congenic BL6JRcc.BL6J‐*Nnt* ^*C57BL/6J*^/Wuhap (*Nnt* ^*Δ*^) mice, harboring the *Nnt* ^*C57BL/6J*^ deficiency, and B6JRcc(B6J)‐*Nnt* ^+^/Wuhap (*Nnt* ^*WT*^) mice, serving as wild‐type controls, were used in this study. These mouse strains were bred and used in our lab to study the metabolic effects of NNT deficiency (Song et al., 2025; Sun et al., 2022).

### 2.2 Experimental overview

All experiments were initially focused on female mice, motivated by the historical underrepresentation of female models in metabolic literature; key experiments were subsequently extended to male mice due to established sex differences in glucose handling. After weaning, mice were group‐housed under controlled environmental conditions (23°C ± 1°C, 12 h/12 h light–dark cycle, 50% ± 10% relative humidity) and provided with ad libitum access to a standard chow diet (Teklad 2920, Inotiv, USA) and water. One month prior to reaching the target ages (6 or 24 months), healthy mice, excluding those with obvious health defects such as tumors or wounds, were transferred to individual housing for environmental acclimatization. The researchers were blinded to the experimental groups when the mice reached the target age, with unblinding performed after dissection. During this period, body weight (BW), body composition (BC) including fat mass (FM) and lean mass (LM), and food intake were monitored weekly using a weighing scale and an Echo MRI Body Composition Analyzer (EchoMRI‐100V, USA).

Experimental approaches were performed on two experimental sets of mice. In the first set (*n* = 13–15), shown in Figure S1a, an oral glucose tolerance test (OGTT) was performed to assess glucose and insulin homeostasis in male and female mice based on previously established protocols (Sun et al., 2022). Three weeks later, BW and BC were measured again, and most of the mice (*n* = 12) were subsequently placed in the Indirect Calorimetry (InCa) PhenoMaster system (TSE Systems, DE) to monitor real‐time metabolic dynamics (see below). Two days later, after a 12‐h fast with free access to water, mice were euthanized by decapitation. Blood glucose levels were measured using a glucometer (FreeStyle Freedom Lite, Abbott Diabetes Care, USA) after decapitation, and trunk blood was collected for insulin analysis, using an Ultra‐Sensitive Mouse Insulin ELISA Kit (Cat. No. 90080, Crystal Chem, NL), according to the manufacturer's instructions. The left *M. gastrocnemius* was isolated and immediately snap‐frozen in liquid nitrogen and preserved at −80°C until Western blot analysis.

In the second set of animals, shown in Figure S1b, 6‐ and 24‐month‐old male and female mice (*n* = 8–12) were subjected to a 5‐h fast, with free access to water. Following this fast, a glucose solution (2 g/kg BW) was administered by oral gavage. Exactly 30 min post‐administration, mice were euthanized by decapitation. Blood glucose levels were measured using a glucometer, and serum was obtained from trunk blood to quantify insulin levels. The left *M. gastrocnemius*, right lobe of the liver and mesenteric white adipose tissue (mWAT) were isolated and placed into cryotubes for Western blot analysis. To preserve tissue morphology for immunofluorescence staining, the right *M. gastrocnemius* was isolated, embedded in CryoCompound (Immunologic, NL), and snap‐frozen in liquid nitrogen‐cooled n‐pentane. All other tissues were snap‐frozen directly in liquid nitrogen. All samples were subsequently stored at −80°C until further use.

### 2.3 Glucose tolerance and insulin resistance

The OGTT was performed as previously described (Costa‐Machado et al., 2023). Briefly, after a 5‐h fast, mice were orally administered glucose (2 g/kg BW). Blood glucose levels were measured using a glucometer (FreeStyle Freedom Lite, Abbott Diabetes Care, USA) from the tail vein at seven time points (*t* = 0, 15, 30, 45, 60, 90, and 120 min). Blood was collected at the first three time points to determine serum insulin concentrations with an Ultra‐Sensitive Mouse Insulin ELISA Kit (Cat. No. 90080, Crystal Chem, NL), according to the manufacturer's instructions. Insulin resistance was evaluated using the Homeostatic Model Assessment index (HOMA‐IR), calculated as: *(fasting glucose [mmol/L] × fasting insulin [mU/L])/22.5*. Additionally, the HOMA of β‐cell function (HOMA‐β) was calculated as: *(20 × fasting insulin [mU/L])/(fasting glucose [mmol/L] − 3.5)* (Matthews et al., 1985). Finally, hepatic insulin resistance index (HIRI) was determined as: *([glucose*_*AUC 0–30*_ *in mmol/L·h] × [insulin*_*AUC 0–30*_ *in pmol/L·h])* (Gijbels et al., 2024; Potter et al., 2023; Vogelzangs et al., 2020).

### 2.4 Indirect calorimetry (InCa)

Energy metabolism was monitored in real‐time using the PhenoMaster system. Before the mice were transferred to InCa cages, BW and BC were determined via a weighing scale and Echo MRI. Mice were randomly assigned to individual InCa cages and acclimated for at least 24 h. Afterwards, a fasting challenge was initiated at 13:00 (water ad libitum), followed by refeeding at 21:00. Oxygen consumption (VO_2_), carbon dioxide production (VCO_2_), Respiratory Exchange Ratio (RER), Energy Expenditure (EE), and physical activity were continuously recorded during this period. To assess the effects of NNT deficiency in the fasted state, metabolic parameters were averaged over the final fasting period (19:00–21:00), a window during which all mice are expected to have reached a fasted state. Carbohydrate (CHO) and fat oxidation rates were calculated from gas exchange data using established formulas as shown below (Miotto & Holloway, 2016), and normalized to LM. *CHO oxidation = 4.585 × VCO*_*2*_ *production (L/min) − 3.226 × VO*_*2*_ *production (L/min)*. And *fat oxidation = 1.695 × VO*_*2*_ *production (L/min) − 1.701 × VCO*_*2*_ *production (L/min)*. The EE was corrected to LM according to a previously validated normalization algorithm (Muller et al., 2021; Speakman et al., 2013; Tschop et al., 2011).

### 2.5 Western blot

The procedure was performed as previously described (Song et al., 2025), with minor modifications. Briefly, 20 mg frozen *M. gastrocnemius* per sample (*n* = 4–8) was placed in an Eppendorf tube with 300 μL cold extraction medium [50 mM Tris‐base, 1 mM EDTA, 1% Glycerol, 0.2% Triton X‐100, 150 mM NaCl, protease inhibitor cocktail (Cat. No. 04693116001, Roche, AT) and phosphatase inhibitor tablet (Cat. No. 04906837001, Roche, AT) pH 7.4], and a metal bead (5 mm). Tissues were subsequently homogenized using Qiagen Tissue Lyser LT (50 Hz, 4 min). Supernatants were collected after centrifugation (10,000 × *g*, 5 min, 4°C). Protein concentrations were determined using the RC DC Protein Assay Kit (Cat. No. 5000116, Bio‐Rad Laboratories, USA). The samples were diluted to 2 mg/mL protein concentration in NuPAGE sample buffer with DTT and 26 μg of protein per lane was loaded in NuPAGE 4%–12% BIS‐TRIS gels (Cat. No. NP0323BOX, Invitrogen, USA). To minimize inter‐blot variability across different groups (*n* = 4 in fasted group and *n* = 8 in glucose‐stimulated group), target bands were excised from different gels according to molecular weight and transferred simultaneously onto a single 0.45 μm PVDF membrane (Cat. No. IPFL85R, EMD Millipore, USA) within one transfer cassette. After protein transfer, the membranes were blocked with intercept blocking buffer (927‐60001, Li‐Cor) for 1 h at room temperature. Then the membranes were incubated with primary antibodies overnight at 4°C. The primary antibodies used were: rabbit anti‐NNT (1:1000, 13442‐2‐AP, Proteintech), rabbit anti‐GAPDH (1:5000, 2118S, Cell Signaling), rabbit anti‐p‐mTOR (Ser2448) (1:1000, 5536S, Cell Signaling), rabbit anti‐mTOR (1:1000, 2983S, Cell Signaling), rabbit anti‐p‐AKT (Ser473) (1:1000, 4060S, Cell Signaling), rabbit anti‐p‐AKT (Thr308) (1:1000, 13038S, Cell Signaling), mouse anti‐AKT2 (1:1000, 5239S, Cell Signaling), rabbit anti‐PI3K p110α (1:2000, 27921‐1‐AP, Proteintech), and rabbit anti‐STAT3 (79D7) (1:2000, 4904T, Cell Signaling). Primary antibodies were detected using an IRDye 800CW‐conjugated goat anti‐mouse secondary antibody (1:5000, 926‐32210, Li‐Cor) or an IRDye 680RD‐conjugated donkey anti‐rabbit secondary antibody (1:5000, 926‐68073, Li‐Cor) and membranes were scanned using an Odyssey scanner (LI‐COR, DE). The labeling intensity was quantified using ImageJ (U.S. National Institutes of Health, USA).

### 2.6 Immunohistochemistry

Methodology to assess sarcolemmal GLUT4 localization and the verification of the GLUT4 antibody were described previously (Bradley et al., 2015). Frozen *M. gastrocnemius* samples from all groups were transversally sectioned under identical conditions at −23°C at a thickness of 5 μm using a Leica cryostat, and mounted onto SuperFrost Plus glass slides (Thermo Fisher Scientific, USA). Sections were post‐fixed in 4% paraformaldehyde (PFA) in 0.1 M phosphate‐buffered saline (PBS) for 15 min at room temperature, followed by three 5‐min washes in 0.01 M Tris‐buffered saline (TBS; pH 7.4). To reduce background fluorescence, sections were incubated in 10 mM TBS‐Glycine (pH 7.4) for 30 min. Sections were permeabilized with 0.5% Triton X‐100 for 10 min and then blocked with 10% normal goat serum (Cat. No. S‐1000, Vector Laboratories, USA) for 30 min. Primary antibodies, specifically rabbit anti‐GLUT4 (1:400; PA5‐23052, Invitrogen) and rat anti‐Laminin‐2α (1:400; ab11576, Abcam) were diluted in 0.01 M TBS containing 0.05% BSAc (final concentration, diluted from a 10% stock), applied to the sections, and incubated overnight at 4°C. Negative controls were incubated with rabbit (1:10,000, Cat. No. I‐1000, Vector Laboratories, USA) and rat IgG (1:10,000, Cat. No. I‐4000, Vector Laboratories, USA) to confirm primary antibody specificity. Following this incubation, the sections were washed and then incubated for 1 h in the dark with secondary antibodies: Alexa Fluor 488‐conjugated goat anti‐rabbit IgG (1:200, Cat. No. A11008, Thermo Fisher, USA) and Alexa Fluor 594‐conjugated goat anti‐rat IgG (1:200, Cat. No. A11007, Thermo Fisher, USA). Nuclei were counterstained with DAPI (1:20,000 diluted in 0.01 M TBS, Cat. No. D9564, Sigma‐Aldrich, USA) for 10 min. Slides were mounted using FluoroMount‐G (Cat. No. 0100‐01, SouthernBiotech, USA) and imaged at room temperature using a Leica widefield microscope (DM6 B, Leica Biosystems, DE) equipped with a 120 W Metal‐Halide light source (Leica EL6000), a digital camera (DFC365 FX) and a 20× objective lens. Image capture was performed using Leica Application Suite X (LAS X) software. For quantitative analysis, two slides were examined per biological sample. Random, non‐overlapping fields of view were imaged across these sections to ensure that a minimum of 200 myofibers were quantified per biological sample. All imaging parameters were kept consistent across samples, and no non‐linear adjustments were made to image intensity or contrast prior to analysis. Colocalization of GLUT4 and Laminin alpha 2 (Laminin‐2α) was analyzed using a custom Fiji ImageJ script via the Coloc 2 plugin (Dunn et al., 2011; Schindelin et al., 2012). The script was generated with the assistance of Google Gemini (Version 3.1 pro) from February 2026 to March 2026; the authors thoroughly verified the code for accuracy and take full responsibility for the data integrity and final results. Manders' Colocalization Coefficients (MCC) was calculated for all images using a Costes automated threshold to eliminate background bias and ensure statistical validity (Costes et al., 2004; Li et al., 2022; Manders et al., 1993).

### 2.7 Statistics

All statistical analyses were performed using GraphPad Prism 10.6.1 (GraphPad Software Inc., USA). The normal distribution and homogeneity of variance were assessed using the Shapiro–Wilk and Brown‐Forsythe tests, respectively. Non‐normally distributed data were log10‐transformed for statistical testing. All figures present raw data, while *p*‐values were derived from the transformed values where applicable. Data shown in bar charts were analyzed using two‐way Analysis of Variance (two‐way ANOVA) followed by Tukey's post hoc test, with genotype and age as fixed factors. For glucose and insulin curves, two‐way repeated measures ANOVA followed by Bonferroni's multiple comparisons test were used. All data are presented as means ± SD. Statistical significance was set at *p* < 0.05.

## 3 RESULTS

### 3.1 Old age, but not NNT deficiency, significantly alters body composition and systemic metabolism in both sexes

To evaluate the impact of aging and NNT deficiency on physiological and metabolic parameters, we compared 6‐month‐old (adult) and 24‐month‐old (old) *Nnt* ^*WT*^ and *Nnt* ^*Δ*^ male and female mice. In males, old mice exhibited significantly higher BW (Figure 1a), FM (Figure 1b), LM (Figure 1c), and weekly food intake (WFI) (Figure 1d) compared to adult mice (*p* < 0.0001). Notably, no significant differences were observed between *Nnt* ^*WT*^ and *Nnt* ^*Δ*^ mice for any of these physiological parameters (*p* > 0.05), and no interaction between age and genotype was detected. To accurately account for this variable, raw EE was normalized to LM (Figure 1e). Subsequent analysis showed that adjusted EE was lower in old males (Figure 1e, *p* < 0.0001) and was unaffected by genotype. Physical activity, measured as total beam breaks during the fasted period, was significantly lower with age in both genotypes (Figure 1f, *p* = 0.0086). Substrate utilization also shifted with age, but was not influenced by genotype. Old mice displayed a significantly lower fasted RER compared to adult mice (Figure 1g, *p* = 0.0008), accompanied by lower fasted CHO oxidation (Figure 1i, *p* < 0.0001), whereas fat oxidation did not differ significantly between age groups (Figure 1h). No genotype effect or interaction was detected for any calorimetry parameter.

**FIGURE 1:**
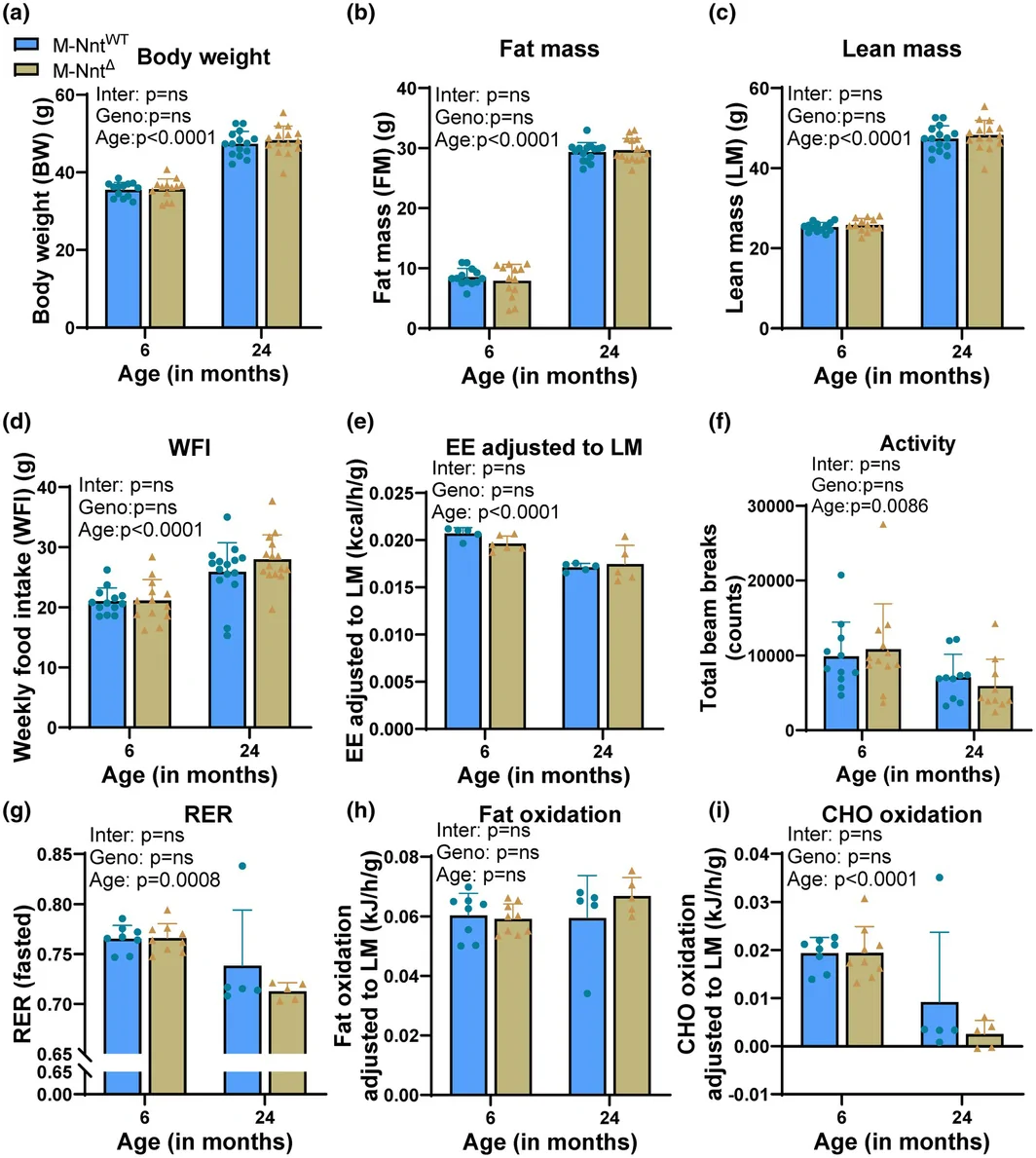
Body composition and systemic metabolism are significantly altered by aging, but remain unaffected by NNT deficiency in male mice. (a–d) Assessment of basic physiological parameters, including body weight (a), fat mass (b), lean mass (LM) (c), and weekly food intake (d) in 6‐ and 24‐month‐old male mice. Legend shown in the upper panel of (a). (e–i) Average indirect calorimetry (InCa) measurements recorded over a 2‐h period (19:00–21:00) immediately following an initial 6‐h fast. Figures show energy expenditure (EE) adjusted to LM (e), spontaneous physical activity quantified by total beam breaks (f), respiratory exchange ratio (RER) (g), fat oxidation corrected to LM (h), and carbohydrate (CHO) oxidation corrected to LM (i) during this 2‐h fasted period. Data are presented as mean ± SD. Statistical differences were evaluated by two‐way ANOVA with Tukey's post hoc test to determine the main effects of age, genotype, and their interaction. The respective *p*‐values (Interaction, Genotype, and Age) are embedded within each panel (ns: not significant). *n* = 13–15 mice per group for (a–d), and *n* = 5–12 mice per group for (e–i).

Females showed the same pattern of age‐related change. Old females had higher BW (Figure 2a), FM (Figure 2b), LM (Figure 2c) and WFI (Figure 2d) than adult females (all *p* < 0.0001), with no genotype effect and no interaction. In contrast to males, lean‐mass‐adjusted EE was preserved with age in females (Figure 2e). Physical activity declined with age (Figure 2f, *p* = 0.0006), and fasted RER was lower in old females (Figure 2g, *p* < 0.0001), and the lower RER was accompanied by both a higher rate of fat oxidation (Figure 2h, *p* < 0.0001) and a lower rate of CHO oxidation (Figure 2i, *p* < 0.0001). As in males, no calorimetry parameter was influenced by genotype or by interaction (all ns). Thus, BW, FM, LM and WFI were higher in old male and female mice, together with reduced activity and a shift away from carbohydrate oxidation, while NNT deficiency did not alter any of these parameters.

**FIGURE 2:**
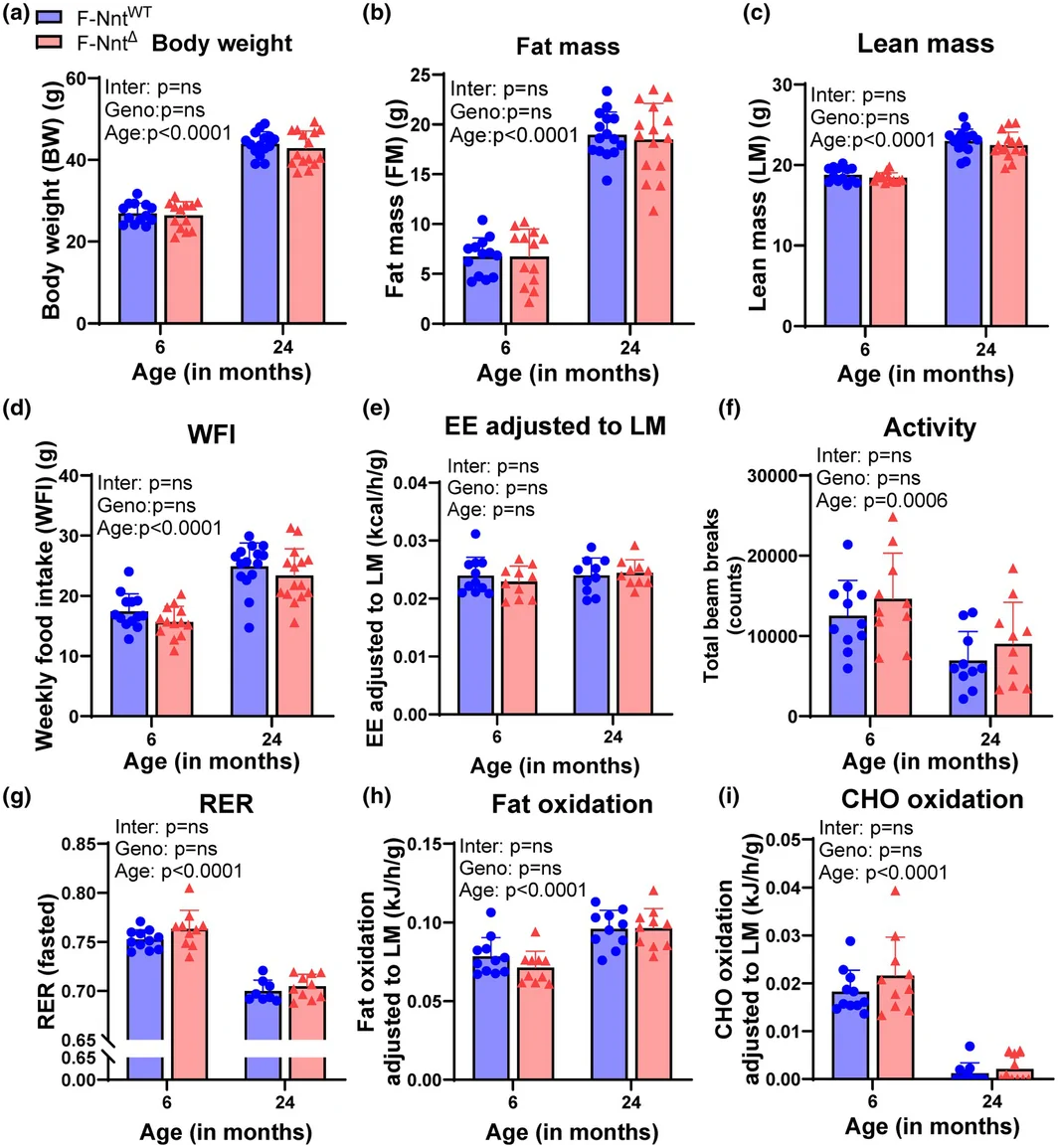
Body composition and systemic metabolism are significantly altered by aging, but remain unaffected by NNT deficiency in female mice. (a–d) Assessment of the basic physiological parameters, including body weight (a), fat mass (b), lean mass (LM) (c), and weekly food intake (d) in 6‐ and 24‐month‐old mice. Legend shown in upper panel of (a). (e–i) Average indirect calorimetry (InCa) measurements recorded during the final 2 h (19:00–21:00) of an 8‐h fasting period. Figures show energy expenditure (EE) adjusted to LM (E), activity quantified by total beam breaks (f), respiratory exchange ratio (RER) (g), fat oxidation corrected to LM (h), and carbohydrate (CHO) oxidation corrected to LM (i). Data are presented as mean ± SD. Statistical differences were evaluated by two‐way ANOVA with Tukey's post hoc tests to determine the main effects of age, genotype and their interaction. The respective *p*‐values (Interaction, Genotype, and Age) are embedded within each panel. *n* = 13–15 mice per group.

### 3.2 NNT deficiency impairs oral glucose tolerance in male mice without altering fasting glycaemia

To assess the impact of NNT deficiency and aging on glucose homeostasis, fasted blood glucose, fasted insulin, and glucose and insulin response were evaluated across male groups. Fasted blood glucose did not differ between age groups or genotypes (Figure 3a), whereas fasted serum insulin (Figure 3b), HOMA‐IR (Figure 3c), and HOMA‐β (Figure 3d) were all higher in old males (all *p* < 0.0001) with no genotype effect and no interaction (all ns). Dynamic glucose handling, by contrast, was affected by genotype. During OGTT, blood glucose was higher in *Nnt* ^*Δ*^ compared to *Nnt* ^*WT*^ males at both 6 months (Figure 3e, *p* < 0.0001) and 24 months (Figure 3f, *p* < 0.0001), with a significant effect of time in both age groups (both *p* < 0.0001) and no interaction effect. The AUC was correspondingly higher in *Nnt* ^*Δ*^ males (Figure 3g, *p* < 0.0001) and lower in old than adult males (age *p* < 0.0001), without an interaction.

**FIGURE 3:**
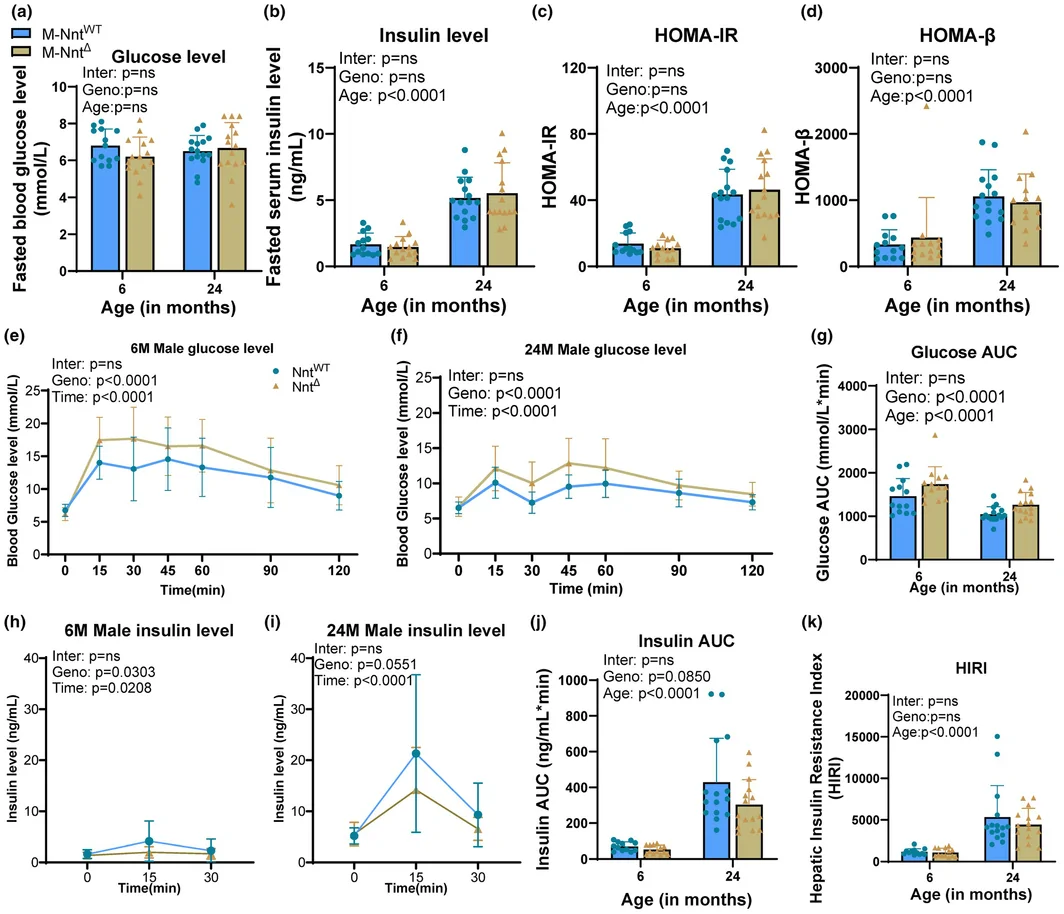
NNT deficiency impairs glucose tolerance in male mice with a modest reduction in the glucose‐stimulated insulin response. (a–d) Evaluation of fasting metabolic parameters in 6‐ and 24‐month‐old male Nnt^WT^ and Nnt^Δ^ mice, including fasted blood glucose levels (a), fasted serum insulin levels (b), the Homeostatic Model Assessment for Insulin Resistance (HOMA‐IR) (c), and β‐cell function (HOMA‐β) (d). (e–g) Blood glucose levels during an oral glucose tolerance test (OGTT) in 6‐month‐old (e) and 24‐month‐old (f) male mice, and the corresponding area under the curve (AUC) analysis (g). (h–j) Serum insulin levels measured at 0, 15, and 30 min during the OGTT for 6‐month‐old (h) and 24‐month‐old (i) male mice, with the calculated insulin AUC (j). (k) The Hepatic Insulin Resistance Index (HIRI). Data are presented as mean ± SD. The data in graphs (e, f, h, i) were analyzed using a two‐way repeated measures ANOVA followed by Bonferroni's multiple comparisons test. The data in graphs (a–d, g, j, k) were analyzed using standard two‐way ANOVA, followed by Tukey's post hoc test to determine the main effects of genotype, age, and their interaction. The respective *p*‐values are embedded directly within each panel. *n* = 13–15 mice per group.

The insulin response to glucose bolus was modestly lower in *Nnt* ^*Δ*^ males. At 6 months, serum insulin over the 0–30 min window showed a genotype main effect (Figure 3h, *p* = 0.030) alongside an effect of time (*p* = 0.021); at 24 months the genotype effect did not reach significance (Figure 3i, *p* = 0.055) although the time effect was strong (*p* < 0.0001). The insulin AUC was significantly higher in old males (Figure 3j, *p* < 0.0001) and did not differ significantly between genotypes (*p* = 0.085). The HIRI was higher with age (Figure 3k, *p* < 0.0001) and unaffected by genotype. Taken together, male *Nnt* ^*Δ*^ mice were glucose intolerant at both ages despite normal fasting glucose levels, and this occurred without a compensatory increase, with a tendency towards a reduction, in the glucose‐stimulated insulin response.

### 3.3 NNT deficiency impairs glucose homeostasis in female mice and lowers the insulin response in old age

To assess the impact of NNT deficiency and aging on glucose homeostasis in female mice, fasted blood glucose, fasted insulin, and derived homeostasis model assessment (HOMA) were evaluated. Age, rather than NNT deficiency, was the primary driver of impairments in fasted blood glucose (Figure 4a, *p* = 0.011), serum insulin (Figure 4b, *p* < 0.0001), insulin resistance (HOMA‐IR) (Figure 4c, *p* < 0.0001) and β‐cell function (HOMA‐β) (Figure 4d, *p* < 0.0001). In contrast to basal states, dynamic glucose regulation was significantly influenced by NNT deficiency. During the glucose tolerance test at 6 months, female *Nnt* ^*Δ*^ mice exhibited a significant impairment in glucose clearance compared to *Nnt* ^*WT*^ mice (Figure 4e, *p* = 0.014). The difference in glucose handling was less robust at 24 months. While the overall genotype effect only displayed a trend (Figure 4f, *p* = 0.060) towards higher glucose levels in *Nnt* ^*Δ*^ mice, there was a significant interaction (*p* = 0.008), indicating that the glucose clearance patterns differed significantly over time. The glucose area under the curve (AUC) was significantly higher in *Nnt* ^*Δ*^ (Figure 4g, *p* = 0.002), and lower in old than adult females (*p* = 0.0005), with a non‐significant interaction (*p* = 0.084).

**FIGURE 4:**
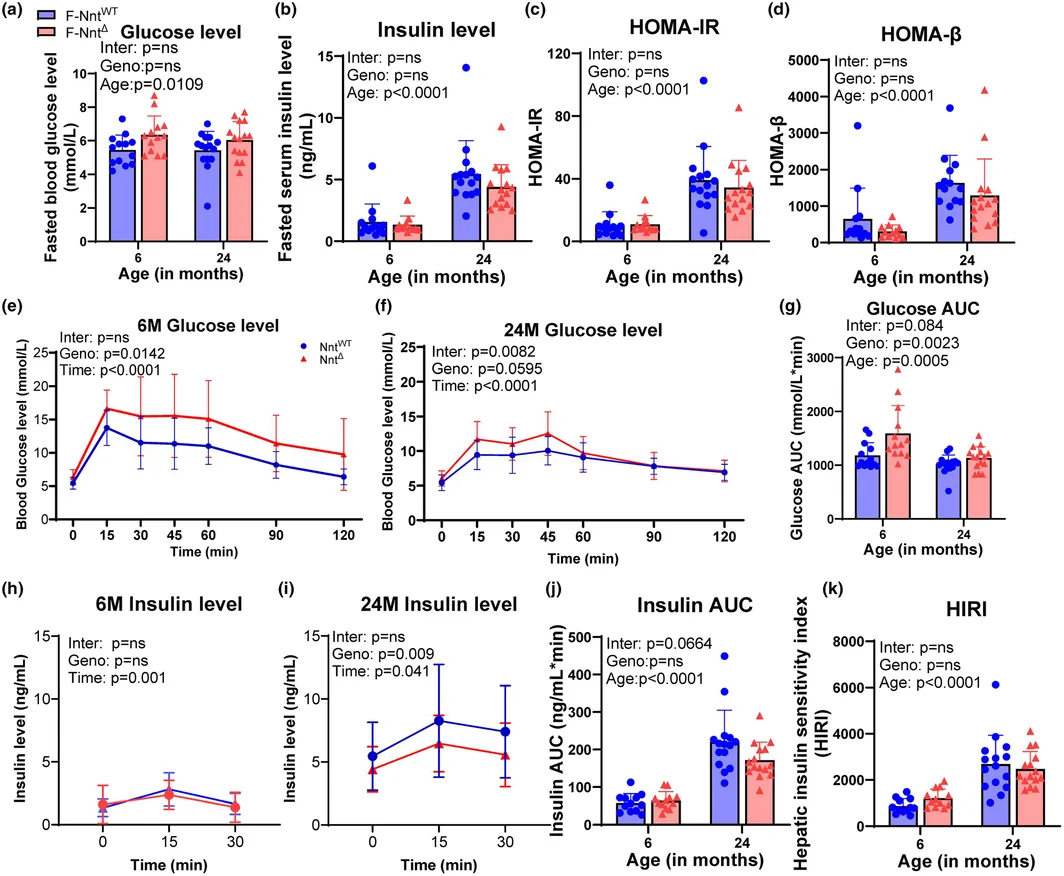
NNT deficiency impairs glucose tolerance in female mice and lowers the insulin response in old age. (a–d) Evaluation of fasting metabolic parameters in 6‐ and 24‐month‐old female Nnt^WT^ and Nnt^Δ^ mice, including fasted blood glucose levels (a), fasted insulin levels (b), the Homeostatic Model Assessment for Insulin Resistance (HOMA‐IR) (c), and β‐cell function (HOMA‐β) (d). (e–g) Blood glucose levels during an oral glucose tolerance test (OGTT) in 6‐month‐old (e) and 24‐month‐old (f) mice, and the corresponding area under the curve (AUC) analysis (g). (h–j) Serum insulin levels measured at 0, 15, and 30 min during the OGTT for 6‐month‐old (h) and 24‐month‐old (i) mice, with the calculated insulin AUC (j). (k) The Hepatic Insulin Resistance Index (HIRI). Data are presented as mean ± SD. The data in graphs (e, f, h, i) were analyzed using a two‐way repeated measures ANOVA followed by Bonferroni's multiple comparisons test. The data in graphs (a–d, g, j, k) were analyzed using standard two‐way ANOVA, followed by Tukey's post hoc test to determine the main effects of genotype, age and their interaction. The respective *p*‐values are embedded directly within each panel. *n* = 13–15 mice per group.

Analysis of insulin levels during the glucose tolerance test (0–30 min) revealed different patterns based on age and genotype. At 6 months, no genotype effects were detected in circulating insulin levels (Figure 4h). However, in old mice, there was a significant main effect of genotype on insulin levels (Figure 4i, *p* = 0.009), whereby insulin was lower in *Nnt* ^*Δ*^ mice. With respect to the insulin AUC, there was a trend towards an interaction effect between age and genotype (Figure 4j, *p* = 0.064). In contrast, the insulin AUC was higher in old mice (Figure 4j, *p* < 0.0001), without any differences between genotypes. The Hepatic Insulin Resistance Index (HIRI) was also higher in old mice (Figure 4k, *p* < 0.0001), but no genotype effect was observed. Taken together, similar to our findings in males, these results demonstrate that oral glucose tolerance is impaired by NNT deficiency and glucose AUC is lower in old, whereas insulin AUC is driven by age not genotype.

### 3.4 NNT deficiency does not alter glucose‐stimulated signaling pathways

To examine acute responses to glucose stimulation, an oral glucose bolus was given to 6‐ and 24‐month‐old male and female mice 30 min before sacrifice (Figure S1b). In males, blood glucose was higher in the glucose‐stimulated condition compared to the fasted state at 6 months of age but was lower at 24 months. While genotype had no effect at 6 months of age, 24‐month‐old *Nnt* ^*Δ*^ males showed significantly higher overall blood glucose than *Nnt* ^*WT*^ mice (Figure S1c, *p* = 0.0059). In females, blood glucose levels were significantly higher after glucose administration compared to fasted levels in both adult and old mice, with no significant genotype differences at 6 months and a trend towards higher levels in *Nnt* ^*Δ*^ females at 24 months (Figure S1d, *p* = 0.060), while insulin levels remained largely unchanged (Figure S1e).

To explore the molecular response to a systemic glucose challenge, we examined key markers of glucose‐stimulated signaling in *M. gastrocnemius* from fasted and glucose‐stimulated mice of both sexes (representative Western blot images are shown in Figure 5a,h). We first confirmed NNT protein deletion in *Nnt* ^*Δ*^ mice, in both fasted and glucose‐stimulated states across ages in males (Figure 5b, *p* < 0.0001) and females (Figure 5i, *p* < 0.0001). Loading control GAPDH levels did not differ among groups in either sex (Figure 5c,j). In males, levels of phosphorylated AKT at Ser473 remained unchanged between age and genotype in both the fasted and glucose‐stimulated states (Figure 5d). Total AKT2 protein levels were significantly higher in old males in the fasted state (Figure 5e, *p* = 0.029), and trended towards being higher after glucose administration (*p* = 0.077), with no genotype effect. Consequently, the ratio of p‐AKT (Ser473)/AKT2 was not affected by age or genotype regardless of the physiological state (Figure 5f). The ratio of the glucose‐stimulated to the fasted signal remained comparable across all male groups, with no effect of genotype, age or their interaction (Figure 5g).

**FIGURE 5:**
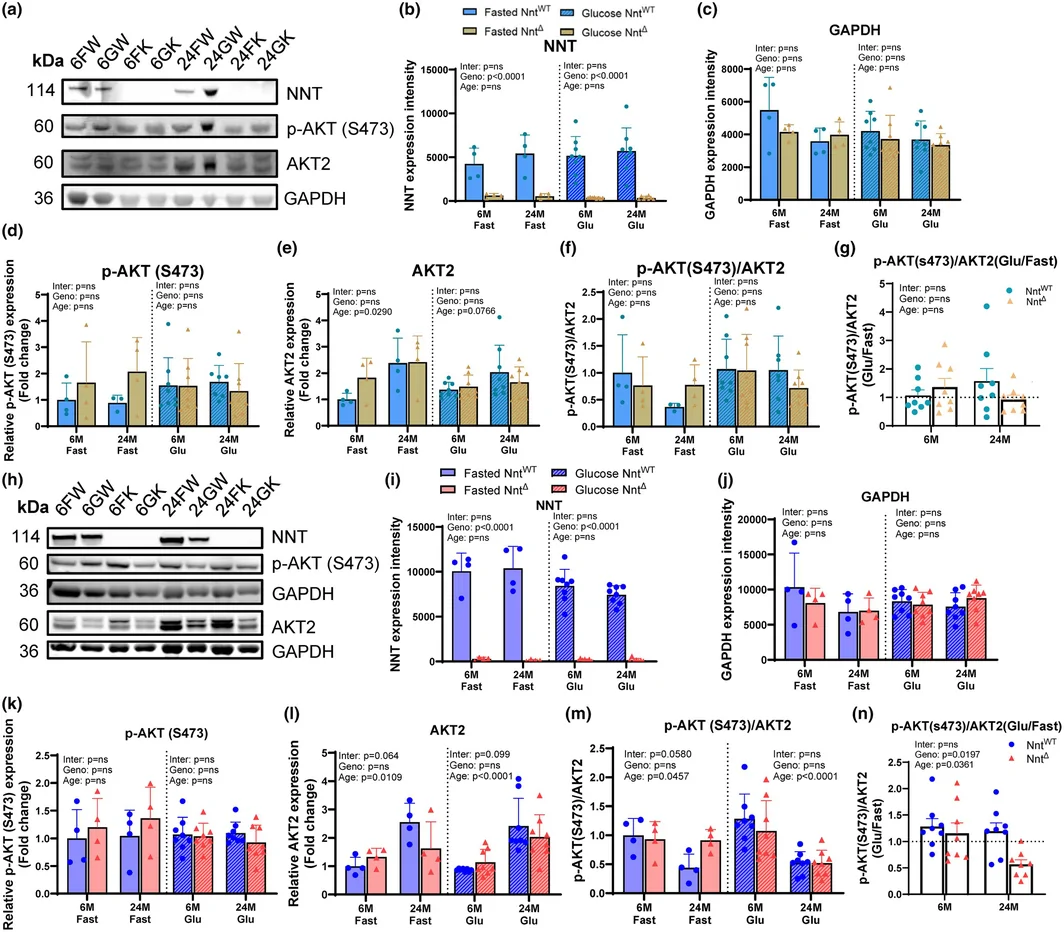
Impact of NNT deficiency on protein content of *M. gastrocnemius* in 6‐ and 24‐month‐old male and female mice. (a–g) Protein content in male mice: (a) Representative Western blot analysis of NNT, phosphorylated AKT (Ser473), total AKT2, and GAPDH in *M. gastrocnemius* lysates. Groups are labeled as follows: 6 and 24 indicate age in months; F, fasted state; G, glucose‐stimulated state; W, Nnt^WT^; and K, Nnt^Δ^. (b, c) Expression intensity of (b) NNT (with the upper panel showing the legend for experimental groups) and (c) loading control GAPDH. (d, e) Quantification of relative phosphorylation and total protein content levels, normalized to the 6‐month‐old Nnt^WT^ group: (d) relative fold‐change of p‐AKT (Ser473) and (e) total AKT2. (f) Calculated ratio of phosphorylated to total protein levels for AKT2 at Ser473. (g) Calculated ratio of glucose‐stimulated to fasted p‐AKT (Ser473)/AKT2 protein content levels. (h–n) Insulin signaling marker levels in female mice: (h) Representative Western blot images of key insulin signaling protein (AKT2), its phosphorylated form (p‐AKT S473), NNT and GAPDH in *M. gastrocnemius* lysates. (i, j) Expression intensity of (i) NNT (with the upper panel showing the legend for experimental groups) and (j) loading control GAPDH. (k, l) Quantification of relative phosphorylation and total protein content levels, normalized to the 6‐month‐old Nnt^WT^ group: (k) relative fold‐change of p‐AKT (Ser473) and (l) total AKT2. (m) Calculated ratio of phosphorylated to total protein levels for AKT2 at Ser473. (n) Calculated ratio of glucose‐stimulated to fasted p‐AKT (Ser473)/AKT2 protein content levels. Full immunoblots with molecular weight markers are available in the Data S1. Data are expressed as mean ± SD. For bar graphs in (b–f) and (i–m), data separated by the dashed line (fasted vs. glucose‐stimulated states, shown as Fast and Glu) were independently analyzed using standard two‐way ANOVA to determine the main effects of genotype, age and their interaction. The respective *p*‐values are embedded directly within each panel. *n* = 4 mice for fasted; *n* = 8 mice for glucose‐stimulated.

Females also showed an absence of a genotype effect with respect to phosphorylated AKT (Ser473) (Figure 5k) and AKT2 (Figure 5l). However, age differences were evident, whereby AKT2 was higher in 24‐month‐old females compared to 6‐month‐old females both when fasted (Figure 5l, *p* = 0.011) and after glucose (*p* < 0.0001). Consequently, the p‐AKT (Ser473)/AKT2 ratio was lower in 24‐month‐old females compared to 6‐month‐old females across both genotypes, both in the fasted state (Figure 5m, *p* = 0.046) and after glucose administration (*p* < 0.0001). Expressing the glucose‐stimulated signal relative to the fasted signal, however, revealed a genotype and an age effect. Specifically, the fold induction of p‐AKT (Ser473)/AKT2 was lower in *Nnt* ^*Δ*^ compared to *Nnt* ^*WT*^ females (Figure 5n, *p* = 0.0197), and lower in old compared to adult females (*p* = 0.0361).

While there were no differences in skeletal muscle p‐AKT (Ser473) between genotypes, we investigated various markers in diverse tissues to establish potential explanatory factors for differences in glucose tolerance. In female *M. gastrocnemius* (Figure S2), with the exception of a genotype‐driven decrease in total mTOR (Figure S2g, *p* = 0.0113), there were no genotype differences in AKT (Thr308), STAT3, and mTOR (Ser2448) phosphorylation either in the fasted state or 30‐min post‐glucose stimulation (Figure S2b–h). Additionally, quantitative analysis of sarcolemmal GLUT4 colocalization 30‐min following glucose ingestion revealed that GLUT4 translocation was not different between female *Nnt* ^*WT*^ and *Nnt* ^*Δ*^ mice at either 6 or 24 months of age (Figure S3a,b). Following confirmation of successful NNT deletion and consistent GAPDH levels (Figure S4a–c), liver and mWAT showed no genotype differences in AKT signaling (Figure S4e–i). However, there were age effects in AKT signaling across all groups, except for p‐AKT (Ser473) levels in the liver (Figure S4d). Specifically, compared to adult mice, old mice exhibited lower p‐AKT (Ser473)/AKT2 ratios in liver and mWAT (Figure S4f,i, *p* ≤ 0.0003) due to higher AKT2 (Figure S4e,h, *p* ≤ 0.0005) and an mWAT‐specific p‐AKT (Ser473) decline (Figure S4g, *p* = 0.0179). Collectively, these data suggest that glucose intolerance in mice lacking NNT occurs without differences in *M. gastrocnemius*, liver, or mWAT glucose handling pathways at 30‐min following glucose ingestion.

## 4 DISCUSSION

In the current study, we evaluated the impact of functional NNT loss on physiological parameters, glucose tolerance and systemic glucose clearance. A major strength of the present study is the utilization of a well‐defined congenic mouse model, which avoided the confounding genetic background effects. Another strength is the analysis of both female and male mice at two ages, which are adult (6‐month‐old) and old (24‐month‐old). Comprehensive in vivo metabolic measurements, including BC, InCa, and food intake, confirmed that baseline physiological parameters and substrate oxidation remained unaffected by NNT deficiency. With respect to glucose tolerance, our data revealed that NNT deficiency significantly impaired glucose tolerance in the adult and old male and female mice. This occurred without any differences in AKT phosphorylation within skeletal muscle, liver, and mWAT, or GLUT4 translocation in skeletal muscle. Altogether, our findings highlight that the impairments in glucose tolerance, as also observed in previous C57Bl6J versus C57Bl6N mouse models, are mediated by lack of NNT, an effect which occurs in both male and female, in adult as well as old mice.

### 4.1 NNT deficiency impairs glucose tolerance

In the present study, NNT deficiency did not significantly alter baseline physiological parameters in either adult or old mice, including BW, FM, LM, and WFI. These findings are critical as they demonstrate that the metabolic phenotypes described hereafter are directly attributable to NNT deficiency, independent of any variations in BC or energy intake. Furthermore, our InCa metabolic analysis revealed that under fasted states, EE, RER, substrate oxidation rates, and physical activity remained unaffected by the loss of NNT in both sexes. In line with our expectations, these data indicate that NNT deficiency does not induce significant alterations in physiological parameters or substrate oxidation, thereby allowing for a clearer interpretation of the specific role of NNT in glucose homeostasis.

The link between NNT and glucose metabolism has been investigated in literature since 2005, with a particular focus on C57BL/6J mice, which harbor a spontaneous *Nnt* mutation (Santos et al., 2017; Toye et al., 2005). A previous study demonstrated that metabolic challenges, such as a high‐fat diet (HFD), exacerbate glucose intolerance and insulin resistance to a much greater extent in NNT‐deficient mice (Nicholson et al., 2010). To evaluate the specific role of NNT without compounding dietary stressors, we maintained mice on a standard chow diet. Our results demonstrate that blood glucose levels, serum insulin levels, and calculated insulin resistance were not significantly affected by NNT deficiency under fasted conditions. However, in both males and females, NNT deficiency significantly impaired glucose clearance during an OGTT. In males, this genotype effect was present at both ages. In females, the effect was significant at 6 months; at 24 months, a significant interaction was observed, with the main effect of genotype narrowly missing significance. Furthermore, we found that NNT deletion resulted in lower insulin levels during the OGTT in 6‐month‐old male mice, 24‐month‐old female mice, and a trend in 24‐month‐old male mice. This is in line with these previous studies showing that glucose intolerance is mainly caused by reduced insulin secretion (Freeman et al., 2006; Toye et al., 2005). However, the impaired glucose clearance observed in 6‐month‐old female mice occurred without a reduction in serum insulin levels following the oral glucose bolus, indicating that NNT deficiency does not alter insulin secretion in adult females. While males show an NNT deficiency effect on insulin levels already in adulthood, females maintain insulin secretion unaffected by NNT deficiency until old age. This extends recent studies demonstrating that some NNT‐deficiency‐associated phenotypes are late‐onset in males (Aguayo‐Mazzucato et al., 2017; Dalla Costa et al., 2025), by highlighting that this delayed onset is distinctly female‐specific on insulin regulation. These findings suggest that NNT deficiency may affect glucose‐stimulated insulin secretion in a sex‐ and age‐dependent manner. However, we did not observe any differences in HOMA‐β between genotypes, providing indirect evidence to suggest that, at least in the fasted state, there were no differences in pancreatic β‐cell function. In addition, it is reported that insulin secretion is not sensitive to lowered levels of NNT; rather, the primary regulation is driven by increased NNT to enhance insulin secretion (Wong et al., 2010). We hypothesized that the impaired glucose handling in mice lacking NNT was mainly driven by tissue‐specific changes in the response to a glucose load. However, despite the impairment in whole‐body glucose tolerance in *Nnt* ^*Δ*^ mice, the phosphorylation of AKT (Ser473 and Thr308), and of mTOR (Ser2448), and the consequent translocation of GLUT4 to the sarcolemma, remained unaffected by NNT deficiency 30 min after a glucose bolus.

It is important to note that although GLUT4 translocation following a glucose load has been shown to peak at 30 min post‐glucose administration (Bradley et al., 2015), and AKT phosphorylation has been detected in primary rat hepatocytes after 30 min of glucose treatment (Stefkovich et al., 2026), we did not observe a higher skeletal muscle AKT phosphorylation in the glucose‐stimulated condition compared to the fasting condition. It remains possible that the tissue‐level responses had returned to baseline at 30 min post‐glucose administration, particularly as insulin levels did not remain elevated at this time‐point. Therefore, we cannot completely exclude the possibility that signaling responses to insulin may have been differentially affected between genotypes. The impaired glucose clearance alongside an unchanged AKT signaling profile suggests that other metabolic pathways may be mediating the observed glucose intolerance. While non‐canonical pathways such as the insulin‐sensitive sorbin and SH3 domain‐containing protein 1 (SORB1, better known as CAP)/Cbl proto‐oncogene (CBL) or protein kinase C (PRKC) could theoretically bypass AKT to alter GLUT4 translocation, these alone may not fully account for the observed phenotype. Taken together, differences in redox response and cellular redox buffering in NNT‐deficient mice could potentially contribute to the observed glucose intolerance, though this warrants further investigation.

### 4.2 Aging induces insulin resistance despite improved glucose tolerance

In this study, our 24‐month‐old mice of both sexes displayed, compared to 6‐month‐old mice, significantly higher BW, FM, LM, and WFI, in combination with reduced activity and RER. In addition, fasted insulin, HOMA‐IR, and HOMA‐β were higher in old groups in both sexes, and fasted glucose levels remained unchanged with age in males but were significantly lower in old females. During the OGTT, old mice of both sexes displayed improved glucose handling, as indicated by a significantly reduced glucose AUC. However, despite this improved glucose clearance, insulin levels were dramatically upregulated in these older mice compared to adult mice. While seemingly counterintuitive, these findings are in line with previous reports indicating that old mice exhibit paradoxically improved glucose tolerance due to reduced hepatic insulin clearance, which maintains higher circulating insulin levels during a glucose challenge (Ehrhardt et al., 2019; Marmentini et al., 2021). This insulin response contrasts with a previous study reporting impaired glucose‐stimulated insulin secretion in old C57BL/6J mice (Aguayo‐Mazzucato et al., 2017). This difference may largely stem from the genetic background. Earlier studies often utilized non‐congenic mouse models, leading to background‐dependent variations in β‐cell secretion or whole‐body metabolism. By contrast, our study employs a congenic mouse model, representing a methodological advantage that eliminates background‐driven variation. At the tissue‐specific level, phosphorylated AKT did not differ between ages. However, total AKT2 protein content was higher in old female mice compared to adult female mice across all tissues tested (skeletal muscle, liver, mWAT), as well as in skeletal muscle in the fasted males. Consequently, the ratio of p‐AKT to total AKT2 was lower in old female mice in all three tissues. In contrast, this ratio did not differ with age in male skeletal muscle. These findings are in contrast with previous work in male C57BL/6J mice, showing that the lower ratio of p‐AKT to total AKT2 results from impaired AKT phosphorylation in *M. soleus* and *extensor digitorum longus*, rather than higher total AKT2 (Frosig et al., 2013). While speculative, the increase in total AKT protein content may represent a compensatory change to counter the age‐related decline in systemic glucose regulation. However, the age‐related changes in glucose tolerance and in tissue protein content occurred independent of the presence or absence of NNT.

We would like to acknowledge specific limitations of our study. While the oral glucose challenge was utilized to model physiological postprandial responses, the 30‐min tissue collection likely missed the endogenous insulin peak observed at 15 min. Moreover, while this approach best reflects responses to endogenous insulin release, an exogenous insulin stimulation would provide a more rigorous indication of tissue‐specific insulin sensitivity. Future studies incorporating insulin tolerance test (ITT) and insulin‐stimulated tissue analyses are required to definitively determine the impact of NNT deficiency on peripheral insulin sensitivity.

In summary, by using a congenic mouse model, this study establishes that NNT is an important modulator of whole‐body glucose homeostasis. We report that glucose tolerance was impaired in both male and female mice lacking NNT, at both adult and old ages. Our findings position NNT as an important target in the maintenance of glucose homeostasis and identify our congenic mouse model as a direct model to study the role of NNT.

## AUTHOR CONTRIBUTIONS

**Jinmeng Yang:** Data curation; formal analysis; funding acquisition; investigation; methodology; project administration; software; validation; visualization. **Jingyi Song:** Conceptualization; investigation; methodology. **Jo‐lene de Deugd:** Investigation; methodology; resources. **Marcel Jaklofsky:** Investigation; methodology; resources. **Georgios Nikolopoulos:** Investigation; methodology; visualization. **Melissa Bekkenkamp‐Grovenstein:** Investigation; methodology; resources. **Claudia Carmone:** Investigation; project administration; resources. **Jaap Keijer:** Conceptualization; methodology; project administration; supervision. **Heather L. Petrick:** Conceptualization; investigation; project administration; supervision. **Sander Grefte:** Conceptualization; methodology; project administration; resources; supervision.

## FUNDING INFORMATION

This work was supported by the China Scholarship Council (CSC) scholarship awarded to Jinmeng Yang (Grant No. 202106300020).

## CONFLICT OF INTEREST STATEMENT

The authors declare that they have no known competing financial interests or personal relationships that could have appeared to influence the work reported in this paper.

## Supporting information


**Figure S1:** Oral glucose significantly increases glucose levels in adult male and female mice. (a) Schematic of the first experimental subset. OGTT was performed. Three weeks later, metabolic parameters were measured by InCa system. Male and female mice were then subject to a 12‐h fast and were sacrificed by decapitation. Blood and skeletal muscle were collected. (b) Schematic of the glucose challenge. Male and female mice were fasted for 5 h, administered an oral glucose bolus, and decapitated 30 min later for skeletal muscle, liver and mWAT collection. (c) Blood glucose levels in male mice, (d) blood glucose levels in female mice, and (e) serum insulin levels in female mice, comparing the fasted baseline (from subset a) and glucose‐stimulated (from subset b) states. Data are expressed as mean ± SD and analyzed by two‐way ANOVA, with *p*‐values embedded in the panels.

**Figure S2:** Protein content remain unchanged between genotypes in 6‐ and 24‐month‐old female mice, except for mTOR level in glucose‐stimulated 24‐month‐old mice. (a) Representative Western blot images of key insulin signaling proteins (mTOR, PI3K p110α, STAT3) and phosphorylated forms (p‐mTOR (Ser2448) and p‐AKT (T308)) in *M. gastrocnemius* lysates. GAPDH was used as the loading control. Groups are labeled as follows: 6 and 24 indicate age in months; F, fasted state; G, glucose‐stimulated state; W, Nnt^WT^; and K, Nnt^Δ^. Full immunoblots with molecular weight markers are available in the Data S1. (b, d–g) Quantification of relative phosphorylation and total protein content levels, normalized to the 6‐month‐old Nnt^WT^ group: (b) p‐AKT (Thr308), (d) PI3K p110α, (e) STAT3, (f) p‐mTOR (Ser2448), and (g) total mTOR. (c, h) Calculated ratios of phosphorylated to total protein levels for (c) AKT2 at Thr308 and (h) mTOR at Ser2448. Data are expressed as mean ± SD. For all bar graphs, data separated by the dashed line (fasted vs. glucose‐stimulated states, shown as Fast and Glu) were independently analyzed using standard two‐way ANOVA to determine the main effects of genotype, age, and their interaction. The respective *p*‐values are embedded directly within each panel. *n* = 4 mice for fasted; *n* = 8 mice for glucose‐stimulated.

**Figure S3:** Aging and NNT deficiency do not alter sarcolemmal GLUT4 localization in female mice. (a) Representative immunofluorescence images of *M. gastrocnemius* cross‐sections from 6‐ and 24‐month‐old female Nnt^WT^ and Nnt^Δ^ glucose‐stimulated mice. Sections were co‐stained with DAPI (nuclei, blue), GLUT4 (green), and the sarcolemma marker Laminin‐2α (red). Merged images display the colocalization of GLUT4 with the sarcolemma. Images were captured at 20× magnification. Scale bars = 100 μm. (b) Quantification of sarcolemmal GLUT4 localization, expressed as the threshold Manders' colocalization coefficient (tM1) representing the fraction of GLUT4 overlapping with Laminin‐2α. Data are presented as mean ± SD. Data were evaluated by standard two‐way ANOVA. The respective *p*‐values are embedded directly within the panel (*n* = 8).

**Figure S4:** The phosphorylation of AKT in the liver and mesenteric white adipose tissue (mWAT) is not affected by NNT deficiency in 6‐ and 24‐month‐old female mice. (a) Representative Western blot images of key insulin signaling protein (AKT2) and its phosphorylated form (p‐AKT S473), and NNT in liver and mWAT. GAPDH was used as the loading control. Groups are labeled as follows: 6 and 24 indicate age in months; W, Nnt^WT^; and K, Nnt^Δ^. Full immunoblots with molecular weight markers are available in the Data S1. (b, c) Quantification of relative protein expression in liver for NNT (b) and GAPDH (c), and there is no significant difference in GAPDH content among groups. (d–f) Quantification of relative phosphorylation, total protein levels and phosphorylation ratios in liver (d–f) and mWAT (g–i), normalized to 6‐month‐old Nnt^WT^ group. Data are expressed as mean ± SD. The respective *p*‐values are embedded directly within each panel. *n* = 8–9 mice per group.

Data S1.

## Data Availability

The data that support the findings of this study are available from the corresponding author upon reasonable request.
