# Supplementary material for: Whole‐body glucose tolerance is impaired in male and female congenic mice lacking nicotinamide nucleotide transhydrogenase (NNT)
Source: Physiol Rep. 2026 Nov 3;14(21):e71167. doi: 10.14814/phy2.71167 (PMC13731841; doi:10.14814/phy2.71167)
Supplement: Supplementary file 1 [file PHY2-14-e71167-s001.docx]

## SUPPLEMENTAL MATERIAL

***Supplemental Figure S1. Oral glucose significantly increases glucose levels in adult male and female mice. (A)*** *Schematic of the first experimental subset. OGTT was performed, three weeks later, metabolic parameters were measured by InCa system. Male and female mice were then subject to a 12-hour fast and were sacrificed by decapitation. Blood and skeletal muscle were collected.* ***(B)*** *Schematic of the glucose challenge. Male and female mice were fasted for 5 hours, administered an oral glucose bolus, and decapitated 30 minutes later for skeletal muscle, liver and mWAT collection.* ***(C)*** *Blood glucose levels in male mice,* ***(D)*** *blood glucose levels in female mice, and* ***(E)*** *serum insulin levels in female mice, comparing the fasted baseline (from subset A) and glucose-stimulated (from subset B) states. Data are expressed as mean ± SD and analyzed by two-way ANOVA, with p-values embedded in the panels.*

***Supplemental Figure S2. Protein content remain unchanged between genotypes in 6- and 24-month-old female mice, except for mTOR level in glucose-stimulated 24-month-old mice. (A)*** *Representative Western blot images of key insulin signaling proteins (mTOR, PI3K p110α, STAT3) and phosphorylated forms (p-mTOR (Ser2448) and p-AKT(T308)) in M. gastrocnemius lysates. GAPDH was used as the loading control. Groups are labeled as follows: 6 and 24 indicate age in months; F, fasted state; G, glucose-stimulated state; W, Nnt^WT^; and K, Nnt^Δ^. Full immunoblots with molecular weight markers are available in the Supplementary Data S1. (B, D–G) Quantification of relative phosphorylation and total protein content levels, normalized to the 6-month-old Nnt^WT^ group:* ***(B)*** *p-AKT (Thr308),* ***(D)*** *PI3K p110α,* ***(E)*** *STAT3,* ***(F)*** *p-mTOR (Ser2448), and* ***(G)*** *total mTOR.* ***(C, H)*** *Calculated ratios of phosphorylated to total protein levels for* ***(C)*** *AKT2 at Thr308 and* ***(H)*** *mTOR at Ser2448. Data are expressed as mean ± SD. For all bar graphs, data separated by the dashed line (fasted vs. glucose-stimulated states, shown as Fast and Glu) were independently analyzed using standard two-way ANOVA to determine the main effects of genotype, age, and their interaction. The respective p-values are embedded directly within each panel. n=4 mice for fasted; n=8 mice for glucose-stimulated.*

***Supplemental Figure S3. Aging and NNT deficiency do not alter sarcolemmal GLUT4 localization in female mice. (A)*** *Representative immunofluorescence images of M. gastrocnemius cross-sections from 6- and 24-month-old female Nnt^WT^ and Nnt^Δ^ glucose-stimulated mice. Sections were co-stained with DAPI (nuclei, blue), GLUT4 (green), and the sarcolemma marker Laminin-2α (red). Merged images display the colocalization of GLUT4 with the sarcolemma. Images were captured at 20x magnification. Scale bars=100 µm.* ***(B)*** *Quantification of sarcolemmal GLUT4 localization, expressed as the threshold Manders' colocalization coefficient (tM1) representing the fraction of GLUT4 overlapping with Laminin-2α. Data are presented as mean ± SD. Data were evaluated by standard two-way ANOVA. The respective p-values are embedded directly within the panel (n=8).*

***Supplemental Figure S4. The phosphorylation of AKT in the liver and mesenteric white adipose tissue (mWAT) is not affected by NNT deficiency in 6- and 24-month-old female mice. (A)*** *Representative Western blot images of key insulin signaling protein (AKT2) and its phosphorylated form (p-AKT S473), and NNT in liver and mWAT. GAPDH was used as the loading control. Groups are labeled as follows: 6 and 24 indicate age in months; W, Nnt^WT^; and K, Nnt^Δ^. Full immunoblots with molecular weight markers are available in the Supplementary Data S1.* ***(B-C)*** *Quantification of relative protein expression in liver for NNT* ***(B)*** *and GAPDH* ***(C)****, and there is no significant difference in GAPDH content among groups.* ***(D–F)*** *Quantification of relative phosphorylation, total protein levels and phosphorylation ratios in liver* ***(D-F)*** *and mWAT* ***(G-I)****, normalized to 6-month-old Nnt^WT^ group.* *Data are expressed as mean ± SD. The respective p-values are embedded directly within each panel. n=8-9 mice per group.*
